# Prospects of Probiotic Adjuvant Drugs in Clinical Treatment

**DOI:** 10.3390/nu14224723

**Published:** 2022-11-09

**Authors:** Zhongyue Ren, Yan Hong, Yalan Huo, Lingling Peng, Huihui Lv, Jiahui Chen, Zhihua Wu, Cuixiang Wan

**Affiliations:** 1 State Key Laboratory of Food Science and Technology, Nanchang University, Nanchang 330047, China; 2 Jiangxi Institution for Drug Control, Nanchang 330024, China; 3 Department of Medicinal Chemistry and Molecular Pharmacology, College of Pharmacy, Purdue University, 575 W Stadium Ave., West Lafayette, IN 47907, USA; 4 Jiangxi-OAI Joint Research Institute, Nanchang University, Nanchang 330047, China

**Keywords:** complementary medicine, drug side effects, intestinal flora, probiotics

## Abstract

In modern society, where new diseases and viruses are constantly emerging, drugs are still the most important means of resistance. However, adverse effects and diminished efficacy remain the leading cause of treatment failure and a major determinant of impaired health-related quality of life for patients. Clinical studies have shown that the disturbance of the gut microbial structure plays a crucial role in the toxic and side effects of drugs. It is well known that probiotics have the ability to maintain the balance of intestinal microecology, which implies their potential as an adjunct to prevent and alleviate the adverse reactions of drugs and to make medicines play a better role. In addition, in the past decade, probiotics have been found to have excellent prevention and alleviation effects in drug toxicity side effects, such as liver injury. In this review, we summarize the development history of probiotics, discuss the impact on drug side effects of probiotics, and propose the underlying mechanisms. Probiotics will be a new star in the world of complementary medicine.

## 1. Introduction

The intestine is not only an important place for human digestion and absorption but is also the largest immune organ, which plays an extremely important role in maintaining normal immune defense function. It provides a good habitat for micro-organisms and serves as the main organ for human metabolism [1]. As the largest and most complex micro-ecosystem in the human body, gut microbes themselves and their metabolites not only regulate human health but also play an important role as a bridge between diet and the host. As Nobel laureate Joshua Lederberg once pointed out, the human body and human symbiotic microbes constitute superorganisms [2], and there has been an increasing amount of research on the effects of gut microbes on human health and their specific mechanisms.

Currently, drug therapy is the most common means of remission in humans [3,4], but some are not particularly effective, or they cause additional damage to the body [5,6]. Many diseases, such as drug-induced organ damage and chemotherapy side effects, limit the application of drugs and reduce the therapeutic effect [7,8]. At the same time, the intestinal flora will also change with the occurrence of disease [9]. Therefore, with the exploration of the microbial world, maintaining the balance of intestinal flora is gradually being recognized as having a pivotal role in solving the problem of drugs.

In this review, we summarize the mechanism of action of probiotics and integrate the evidence to further discuss the clinical application prospects of probiotics as adjuvant drugs.

## 2. Probiotics

Probiotics are defined as live micro-organisms that, when administered in adequate amounts, confer a health benefit on the host [10]. The term probiotic (derived from the Greek word) was mentioned for the first time in the early 1950s by Werner Kollath to define the importance of the active substances that were crucial to the development of healthy life [11]. In 1965, Lilly and Stillwell first used the definition of probiotics to describe the effect of one micro-organism on the growth of other micro-organisms [12]. In 1989, Fuller redefined probiotics as additionally supplemented active micro-organisms that improve the balance of intestinal flora and are beneficial to the health of the host [13]. In 2002, the Food and Agriculture Organization of the United Nations (FAO) and the World Health Organization (WHO) jointly drafted the “*Guidelines for the Evaluation of Food Probiotics*”, which were widely adopted by national management departments, academia and industry.

The microbes used as probiotics represent different types, such as bacteria, yeast or mold. However, there are more common species (Table 1) [14,15,16].

In 1878, Lister first isolated *Lactococcus* from rancid milk [17]. In 1892, Doderlein suggested that lactic acid-producing micro-organisms are beneficial to humans while studying the vagina [18]. In 1899, Henry Tissier was the first to isolate the first strain of *bifidus* from the feces of healthy breastfed infants and found that it was associated with both the frequency and nutrition of infants suffering from diarrhea [19]. In 1905, Stamen Grigorov first discovered and isolated *Lactobacillus bulgaricus* from yogurt [20]. In 1917, Alfred Nissle took a strain of *Escherichia coli* from the feces of soldiers in the First World War and used this strain to treat intestinal infections (by *Salmonella* and *Shigella*) [21]. In 1922, Rettger and Cheplin reported the clinical efficacy of *Lactobacillus acidophilus* yogurt, especially the functional properties of digestion [22]. In recent years, probiotics have gradually become a hot topic in society, and a large number of studies have proved that probiotics have the ability to alleviate and prevent diseases. Xie et al. proved that a probiotic mixture protected dopamine neurons and further attenuated the deterioration of motor dysfunctions in Parkinson’s disease mice [23]. Tan et al. found *Lactobacillus plantarum* DR7 improved the gut microbiota profile of a Drosophila melanogaster Alzheimer’s disease model and alleviated neurodegeneration in the eye [24]. Clinical research showed that probiotics could be a supplementary therapeutic approach in type 2 diabetes mellitus patients to improve dyslipidemia and promote better metabolic control [25]. The infants who received *Lactobacillus rhamnosus* HN001 daily from birth until the age of 2 years were effective in reducing the incidence of eczema at 2, 4 and 6 years of age and atopic allergy at 6 years of age [26]. Besides these results, probiotics also played a key role in the treatment of oral diseases [27]. *Streptococcus dentisani* 7746 inhibited periodontal pathogens through competition, adhesion and displacement mechanisms [28]. Clinical index (plaque and gingival bleeding) and microbiological parameters improved in patients with chronic periodontitis following a subgingival delivery of probiotics and a probiotic mouthwash and oral administration of *Lactobacillus salivarius* and *Lactobacillus reuteri* [29]. Rodríguez et al. conducted a triple-blind, randomized, placebo-controlled trial to compare the prevalence of dental caries in preschool children with milk supplemented with the probiotic *Lactobacillus* and standard milk [30]. A total of 261 children, aged 2–3 years, were randomly assigned to two parallel groups. The experimental group received milk supplemented with *L. rhamnosus* SP1, while the control group received standard milk. At baseline, there was no difference in caries prevalence between the two groups. After 10 months of intervention, the prevalence of dental caries in the probiotic group was 11.4% lower than in the control group. The percentage of new individuals with cavitary lesions in the control group (24.3%) was significantly higher than in the probiotic group (9.7%). These studies have developed new avenues for the application of probiotics. However, the mechanism of probiotics is still incomplete, which limits the exploration and further development of probiotics.

## 3. Probiotic Adjuvant Drugs in Human Disease

In recent years, under the influence of various factors, such as environmental pollution [31,32], dietary shifts [33], unhealthy lifestyle [34], etc., the types and probability of human diseases, including cancer [35], diabetes [36], cardiovascular disease [37], etc., have greatly increased. Accompanied by the gradually rising medicine-using frequency, side effects and a weakening of efficacy have become increasingly serious problems. Meanwhile, intestinal flora has been proven to change with variable diseases, such as drug-induced organ damage, chemotherapy, etc. Gut flora, however, might be the potential target for solving the serious problems of drugs due to the powerful effect of probiotics on maintaining the balance of intestinal flora. Here, we will successively discuss the effect of probiotics on typical drug-induced injury and the effect of improving drug efficacy.

### 3.1. Probiotic and Drug-Induced Liver Injury

The liver is the most important organ of the body’s metabolism and the main target of drug damage. Drug-induced liver injury (DILI) refers to the liver damage caused by the drug itself or its metabolites during the use of the drug [38]. The mild cases can be recovered after stopping the drug, and the severe cases can cause irreversible liver damage or even death. The researchers found that 13.9 to 24 people per 100,000 people suffer from DILI [39]. The two main categories of DILI are dose-dependent common drug-induced and dose-independent specific drug-induced, the latter being relatively rare [40]. Liver damage from acetaminophen (APAP) is often associated with overdose [41]. APAP can be used for the treatment of cold and fever, arthralgia, neuralgia, migraine, cancer pain and postoperative pain relief, etc. It is one of the main drugs for analgesia and fever treatment [42]. In addition to APAP, many drugs have also been associated with DILI. Antibiotics and antimicrobials accounted for more than 46% of all DILI cases in the Drug-Induced Liver Injury Network (DILIN) cohort from the United States. As an important safety issue, DILI is one of the most common reasons for stopping drug development and withdrawing drugs from the market before and after clinical studies. To increase awareness of DILI and facilitate clinical treatment and improvement, the American College of Gastroenterology developed practice guidelines for the diagnosis and treatment of idiopathic DILI using an evidence-based approach.

Based on numerous studies, the gut microbiota has been shown to modulate many extra-intestinal organ diseases, including liver damage. For example, during CCl_4_-induced liver fibrosis, gut bacteria translocate to the liver and promote liver inflammation. In addition, saturated fatty acids, produced by gut microbes, are protective against alcohol-induced liver damage. Probiotics have long been shown to modulate gut flora, so it is not surprising that probiotics can prevent or alleviate the liver damage caused by drugs. Table 2 shows that when probiotics are used to prevent or mitigate DILI, the abundance of beneficial bacteria, such as *Lactobacillus*, *Bifidobacterium*, *Clostridiales*, etc., in the gut increases. Most of these beneficial bacteria are closely related to short-chain fatty acids (SCFAs), so it is reasonable to speculate that the mechanism of probiotics against drug hepatotoxicity may be related to the secretion of the SCFAs promoted by regulating intestinal flora.

### 3.2. Probiotic and Drug-Induced Kidney Injury

The kidneys are organs that perform many essential functions in the body, including removing endogenous waste products, controlling volume status, maintaining electrolyte and acid-base balance, and endocrine functions [50]. Renal toxicity can occur due to high local concentrations of drugs and toxins in the kidneys and/or their conversion to active metabolites [51]. In fact, drug-induced kidney injury (DIKI) is a serious problem in clinical practice, accounting for 19–26% of acute kidney injury (AKI) cases in hospitalized patients [52]. At present, there are still no therapeutic strategies and candidate drugs to deal with the increasing prevalence of DIKI.

Recently, studies have illustrated that intestinal flora might hold a relationship with DIKI. CCl_4_ also disrupted the intestinal flora balance of mice while causing DIKI, mainly by increasing the relative abundance of *Firmicutes* and decreasing the relative abundance of *Bacteroidetes* and *Proteobacteria* [53]. Cisplatin injection also resulted in CIKI and also significantly increased the relative abundance of *Bacteroidetes* and *Deferribacteres*, decreased the relative abundance of *Firmicutes*, and decreased the ratio of *Firmicutes*/*Bacteroidetes*. Additionally, it raised the relative abundance of *Alloprevotella*, *Bacteroides*, *Bilophila* and *Sutterellaceae* and decreased the *Blautia* and *Oscillibacter* levels in feces [54].

The gut, the microbiota and the kidney are closely related. The “gut-kidney axis” is formed between the gut and the kidney through two pathways of metabolism dependence and immunity, and the gut plays a fundamental role in kidney disease [55]. Probiotics, which are recognized to modulate the gut microbiota, are considered to be a potential new candidate or targeted dietary pattern for the safe prevention and treatment of DIKI. Li et al. found that *L. salivarius* BP121 alleviated intestinal dysbiosis by increasing the abundance of the beneficial intestinal flora, *Lactobacillus* spp., which in turn inhibited the secretion of uremic toxins (such as indoxyl sulfate and *p*-cresol sulfate) and increased the concentration of short-chain fatty acids in feces, thereby preventing cisplatin-induced DIKI [56]. A commercial probiotic formula, PROBIO, comprising *L. acidophilus*, *L. bulgaricus*, *Bifidobacterium bifidum* and fructooligosaccharide as a prebiotic, was proven to resist the nephrotoxicity and oxidative stress, which were induced by gentamicin [57]. *L. rhamnosus* GKLC1 inhibits cisplatin-induced kidney injury by reducing inflammation and inhibiting apoptosis through the MAPKs/NF-ĸB/COX-2 pathway [58]. *L. plantarum* AD3 could regulate the host’s antioxidant system and alleviate the nephrotoxic effect of acetaminophen by increasing the proportion of lactic acid bacteria in the intestinal flora [59]. Probiotics may be a special supplementary drug essential in the future because they can intervene in the gut–kidney axis by regulating the intestinal flora, thereby preventing and alleviating DIKI.

### 3.3. Probiotic and Chemotherapy

Chemotherapy is a broad term for a class of drugs that nonspecifically block cell replication and cause cell death. At present, chemotherapy is still the main treatment method for tumors and is widely used in the treatment of tumors. While chemotherapy generally inhibits cancer development, it can lead to increased mortality in patients through its devastating short- and long-term side effects, including weight loss, skeletal muscle loss, fatigue and psychiatric comorbidities. Local anesthetics, analgesics and antibiotics are often used clinically to deal with chemotherapy-induced bowel damage. However, these treatments only provide short-term and temporary relief from chemotherapy-induced ailments and do not reduce the duration and severity of this pathological condition. Chemotherapy side effects are mostly related to gastrointestinal discomfort: diarrhea, disruption of the gut microbiome and damage to the intestinal epithelium. The mechanisms underlying the side effects of chemotherapeutic drugs are very complex and have not yet been fully studied. In recent years, studies have found that chemotherapy, in addition to relieving the disease, also changes the intestinal flora of patients [60,61,62,63]. Moreover, some chemotherapeutic drugs interact with the microbiome components, resulting in drug safety and/or efficacy changes. For example, irinotecan (CPT-11), a commonly used oncology drug, can also cause severe diarrhea while being treated. Some researchers have observed that CPT-11 treatment altered the intestinal flora and promoted the colonization of pathogenic bacteria that can produce β-glucuronidase, such as *E. coli*, *Staphylococcus* and *Clostridium*, and then lead to enterotoxicity [64,65]. Based on these findings, understanding the impact of the microbiome on the safety and efficacy of cancer treatments may improve treatment outcomes. Probiotics, recognized as safe bacteria that modulate the gut microbiota, also play a positive role in alleviating chemotherapy’s side effects. As shown in Table 3, in the past decade, probiotics have been found to have a relieving effect on traditional chemotherapy side effects (cognitive impairment, colitis, nerve pain, etc.), and most of the relief is accompanied by changes in the intestinal flora. Thus, we speculate that probiotics can restore the intestinal flora destroyed by chemotherapy, protect the intestinal barrier, and reduce intestinal inflammation, so as to help patients relieve the discomfort caused by chemotherapy, strengthen their compliance, and obtain better treatment effects.

### 3.4. Probiotics as Engineered Carriers to Improve Drug Efficacy

In recent years, it has become a trend to improve diseases through technical means at the genetic level, and probiotics as engineering carriers have also been proven by many practices. *Lactobacillus*, as the main source of probiotics, contains a large number of CRISPR-Cas systems, which are beneficial for gene manipulation [75]. The CRISPR-Cas system can be used to enhance the tolerance of probiotics to environmental stress and enhance their adaptation to the intestinal environment. *Lactobacillus* modified by the CRISPR-Cas system can be used as a delivery system for biological and drug therapies [76].

Considering that antibiotics can also damage the normal flora of the human body while treating the disease, Cubillos-Ruiz et al. selected a strain of *Lactococcus lactis* to engineer an intestinal barrier of β-lactamas, which are widely used antibiotics, to protect the local gut microbiota from antibiotic damage [77]. In order to treat colitis, a disease with a long course, easy recurrence and the risk of colon cancer, Yan et al. engineered *E. coli* Nissle 1917 (EcN) to overexpress 3-hydroxybutyrate (3HB) [78]. It can colonize the intestinal tract, use food as raw material to synthesize 3HB, and the 3HB is directly released in situ, which reduces the off-target effect of the drug and makes the drug delivery more effective. Its efficacy is far stronger than that of EcN and 3HB alone. He et al. found that the engineered probiotic, EcN, could specifically deliver the angiogenesis inhibitor Tum-5 and tumor suppressor p53 to anaerobic tumor areas [79]. This treatment method can significantly inhibit the growth of transplanted tumors in nude mice with high efficiency and safety. Chung et al. constructed an engineered probiotic based on *Pediococcus pentosaceus* to carry the gene encoding the therapeutic protein, P8, fused with a secretory signal peptide so that it can inhibit colorectal cancer and promote the restoration of intestinal flora balance [80]. The EcN-engineered probiotic system, constructed by Gurbatri et al., can colonize and enrich tumors and release nanobodies that block the immune checkpoints PD-L1 and CTLA-4, thereby stimulating anti-tumor immune responses, which can not only effectively reduce local tumors but also inhibit tumor metastasis [81]. In addition, these bacteria can also be used in combination with the same type of engineered bacteria expressing cytokines with immunostimulatory effects to further enhance the anti-tumor response, which can significantly enhance the immunotherapy effect on “cold” tumors. Canale et al. constructed an engineered probiotic based on EcN, which colonized tumors and continuously converted the nitrogen–hydrogen compounds accumulated in tumors into L-arginine [82]. Colonization of these bacteria in tumors increases the intratumoral L-arginine concentration and promotes effector T-cell function, thereby synergistically enhancing the efficacy of the anti-PD-L1 therapy and forming long-lasting anti-tumor immunity. Light-activated functional bacteria have unique light absorption and fluorescence properties, strong photothermal conversion abilities, high biocompatibility, excellent tumor selectivity and strong anticancer efficacy. In addition to bacterial tumor targeting, light-induced near-infrared fluorescence (FL) of functional bacteria facilitates clear fluorescent tumor visualization. By engineering *B. bifidum* (BB; NBRC 100015) to synthesize the nanoparticle-functionalized bacteria for photothermal cancer immunotherapy, mouse colorectal cancer cells can be efficiently eliminated with the help of an immune response [83]. Based on these achievements, probiotics have been confirmed as engineering carriers to exert more efficient functions than conventional treatment methods by directly producing or efficiently delivering beneficial metabolites to the affected area, secreting antibodies to activate the immune system, and protecting the gut flora. Engineered probiotics may become the mainstream of future medicine.

## 4. The Mechanism of Probiotics as Adjunctive Drugs

### 4.1. Antioxidant Effect

Free radicals are usually oxygen-containing molecules produced during the body’s metabolism, and they contain an unpaired electron, which allows them to attack molecules, cells or tissues in the body. The human body has its own antioxidant defense mechanism to control free radicals. However, when the body cannot effectively process and scavenge free radicals, excess free radicals will disrupt the body’s natural repair system, causing oxidative stress and damage to the body. Some drugs, such as CCl_4_, APAP, etc., induce body damage by inducing oxidative stress. When CCl_4_ enters the hepatocytes, it is activated by the cytochrome P450 enzyme metabolism to generate trichloromethyl free radicals and trichloromethyl peroxide. These free radicals covalently bind to phospholipid molecules on the hepatocyte membrane, endoplasmic reticulum and mitochondria, triggering lipid peroxidation to damage the membrane structure and function. Meanwhile, these free radicals inhibit the activity of calcium pumps on the cell and mitochondrial membranes, causing a large amount of Ca^2+^ influx. The increase of intracellular Ca^2+^ can activate calpain, induce xanthine oxidation, and generate more free radicals, both of which lead to the degeneration and necrosis of liver cells and eventually to DILI. Probiotic strains have certain antioxidant capacity, like scavenging hydroxyl radicals and superoxide anions and producing antioxidants. This ability mainly relies on the following possible mechanisms:

#### 4.1.1. Metal Ion Chelating Ability

Sequestering agents can trap metal ions and prevent metal ions from participating in catalytic oxidation reactions. Studies have found that *S. thermophilus* and *L. casei* have a strong chelating ability to ferrous ions and copper ions [84,85], and the cytoplasmic extract of *Lactobacillus helveticus* can also chelate ferrous ions well [86].

#### 4.1.2. Antioxidant Enzyme System

Probiotics have their own antioxidant enzyme systems. One of the most well-known enzymes is superoxide dismutase (SOD). Superoxide is one of the most abundant reactive oxygen species (ROS) produced by mitochondria. SOD is responsible for catalyzing the decomposition of superoxide into hydrogen peroxide and water and is the main regulator of ROS levels.

Bacteria can use manganese superoxide dismutase (MnSOD), while mammals can use intracellular and extracellular copper–zinc superoxide dismutase (CuZnSOD) and mitochondrial MnSOD, which is evolutionarily similar to bacterial MnSOD. Dismutase is closely related. For example, some probiotics, such as *Lactobacillus fermentum*, have been found to express MnSOD to combat oxidative stress [87].

#### 4.1.3. Antioxidant Metabolites

Probiotics can produce various metabolites with antioxidant activity, such as glutathione, butyrate and folic acid. For example, supplementation with specific probiotics can increase serum vitamin B_12_ and folate levels, increase the total vitamin B_1_ intake, and also increase glutathione levels and glutathione biosynthesis to reduce oxidative stress [88,89,90]. Glutathione scavenges free radicals, such as hydrogen peroxide, hydroxyl radicals and peroxynitrite, mainly by cooperating with selenium-dependent glutathione peroxidase. Studies have found that *L. plantarum* can increase the level of glutathione in the liver tissue of mice with acute liver injury [91]. Butyric acid is a short-chain fatty acid that can be produced by the fermentation of dietary fiber by gut bacteria. One study finds that butyrate-producing *C. butyricum* induces antioxidant enzymes to inhibit hepatic oxidative stress in rats with nonalcoholic fatty liver disease [92].

#### 4.1.4. Enzymes That Regulate Free Radicals

Oxidative stress results from increased ROS or decreased free radical scavenging levels. Reactive oxygen radicals are generated by some enzymatic reactions and chemical processes. Researchers found that probiotics can reduce the expression of ROS-generating enzymes, including NAPDH oxidase (NOX), cyclooxygenase, and cytochrome P_450_ enzymes, etc. [93]. A mixture of probiotics, including *L. fermentum* CECT5716, *Lactobacillus coryniformis* CECT5711 and *Lactobacillus gasseri* CECT5714, reduced NOX activity and NOX-1 and NOX-4 mRNA expression in spontaneously hypertensive rats [94]. *B. amyloliquefaciens* SC06 treatment reduced NOX activity and p47phox expression to alleviate the H_2_O_2_-induced oxidative stress in intestinal porcine epithelial cells [95].

### 4.2. Regulating the Intestinal Microbiota

Intestinal flora refers to the normal micro-organisms in the human intestinal tract. The intestinal bacteria that inhabit the human gut count for more than 100 trillion microbial cells (~4 × 10^13^) and can affect the human body in a variety of ways. For example, it can affect the development and health of the human body by regulating the production and absorption of nutrients. It can synthesize a variety of vitamins necessary for human growth and development and can also use protein residues to synthesize essential amino acids, participate in the metabolism of carbohydrates and proteins, and promote the absorption of mineral elements. Therefore, the gut microbiota is closely related to the body’s metabolism. For this reason, dramatic changes in the composition and function of intestinal micro-organisms, defined as gut microbiota dysbiosis, are associated with gastroenteric disorders, as well as neurologic, respiratory, metabolic, and hepatic disorders, to the point that some investigators have referred to it as an “extra organ” of the host [96].

It is well documented that probiotics can support good health by modulating gut flora. The *B. lactis* Probio-M8 powder can promote the efficacy of the asthma drug Symbicort Turbuhaler by increasing the abundance of *B. animalis*, *B. longum* and other beneficial bacteria in the gut [97]. Probio-M8 can also regulate intestinal flora and affect intestinal metabolism, thereby alleviating depression and anxiety in patients with coronary heart disease through the gut–brain axis, and act as an adjuvant drug to enhance the effect of conventional treatment [98]. The ability to regulate intestinal microbiota may be related to the biological snatch effect of probiotics. The intestinal microflora of normal animals is dominated by anaerobic bacteria, but this situation will change after an illness. After entering the digestive tract, aerobic probiotics can consume oxygen in the intestine during the growth and reproduction process, forming an anaerobic environment in the local area. This is conducive to the growth of anaerobic micro-organisms, thereby restoring the imbalanced flora to a normal state so as to achieve the purposes of disease prevention, treatment, immunity enhancement and growth promotion. The biological snatch effect of probiotics can significantly reduce the number of pathogenic aerobic bacteria and facultative anaerobic bacteria [99]. Moreover, probiotics also can inhibit the growth of pathogenic bacteria through their metabolites, such as producing organic acids, lowering the intestinal pH or producing hydrogen peroxide and natural antibiotics, reducing the production of toxic substances, such as ammonia and amines in the intestine. Tejero-Sariñena et al. showed that the organic acids produced by different probiotic strains reduced the growth of potentially pathogenic micro-organisms [100].

Furthermore, after changing the intestinal flora, the metabolites of the intestinal flora will also change accordingly, which is also the mechanism by which the probiotics act as adjuvant drugs. Short-chain fatty acids (SCFAs), indole derivatives, vitamins, etc., have been proven to have positive effects on the body through a large number of physiological and clinical studies [101,102]. Therefore, we can speculate that probiotics modulate the gut microbiota, which in turn affects intestinal metabolism, preventing or alleviating the side effects of drugs by promoting the production of beneficial metabolites.

### 4.3. Protecting the Intestinal Barrier

The intestinal barrier is the first defense to prevent pathogenic micro-organisms and food allergens from entering the human intestine, ensuring intestinal health. The intestinal barrier can maintain the integrity of the intestinal epithelial cells, thereby exerting the protective effect of the intestinal epithelial cells (IECs) on living organisms. Once this barrier function is disrupted, bacteria and foodborne antigens can easily reach the submucosa, triggering an inflammatory response that can lead to intestinal disorders (such as IBD). Probiotics have been proven to contain a protection ability of the intestinal barrier. At first, the invasion of a large number of exogenous pathogens or the imbalance of the intestinal flora of the body can cause intestinal mucosal damage by destroying the microbial barrier. Zeng et al. found that probiotics can form a microbial barrier to prevent pathogenic bacteria from damaging the epithelial cells [103]. Probiotics can also promote the formation of a mucus layer. The intestinal mucus layer is mainly composed of mucin (MUC) and intestinal trefoil factor (TFF), which is secreted by goblet cells [104]. Probiotics can induce IECs to secrete a large amount of MUC to prevent contact between the intestinal mucosa and pathogenic bacteria, inhibit the adhesion of pathogenic bacteria to IECs, and thus inhibit the colonization of pathogenic bacteria in the intestinal wall [105]. MUC can also specifically bind to pathogenic bacteria, accelerate intestinal peristalsis, and excrete pathogenic bacteria from the body. Probiotics can also induce IECs to secrete TFF and accelerate the repair of the damaged intestinal mucosa. TFF can accelerate damage repair by promoting the movement of epithelial cells, maintaining the integrity of the intestinal mucosa, and can also combine with MUC to form a gel to enhance the defense function of the intestinal mucosa. Tight junction proteins (TJs) are the key proteins that connect cell gaps and regulate the permeability of intestinal mucosa, which can prevent pathogenic bacteria, toxins and antigenic substances in the intestine from infiltrating into the deep intestinal tissue and blood circulation to maintain the health of the body. Probiotics can induce the expression of ZO-1, promote the phosphorylation of occludin and claudin, reduce cell permeability by enhancing the assembly process of intercellular tight junctions, and protect the intestinal mucosal barrier [106]. In addition, probiotics can also prevent the destruction of TJs by pathogenic bacteria, pro-inflammatory factors, endotoxins and other substances by promoting the secretion of anti-inflammatory cytokines and inhibiting the secretion of inflammatory mediators, thereby inhibiting the intestinal mucosal damage mediated by the cytokine signaling pathway, and restoring the integrity of the intestinal mucosal barrier [107]. Probiotics can also stimulate the intestinal mucosal immune response and improve intestinal immunity and disease resistance by promoting the differentiation and maturation of the T lymphocytes and B lymphocytes to resist infection, inflammation and other diseases. Inhibition of abnormal apoptosis of IECs is also an effective measure of probiotics to maintain the intestinal barrier [108].

### 4.4. Modulation of the Immune System

Probiotics can modulate the immune system by interacting with epithelial cells, dendritic cells (DC), macrophages, and lymphocytes [109]. The immune system is divided into innate and adaptive systems. The adaptive immune response relies on B and T lymphocytes that bind to specific antigens [110]. Numerous studies have shown that most DILI is mediated by the adaptive immune system, in which the cytotoxic CD8^+^ T cells play a prominent role [111]. For example, halothane, which is used as an anesthetic, often causes the abnormal expression of IL-6 and IL-1β, leading to fever. CPT-11 mainly inhibits the activity of topoisomerase I, required for cell growth by metabolizing SN-38, thereby inhibiting the proliferation of tumor cells. However, the indiscriminate effects of SN-38 can also lead to the suppression of the immune system, such as the infiltration of CD8^+^ T lymphocytes [112]. Therefore, when probiotics have been shown to modulate immune responses and induce Treg development, they can naturally assist a large number of drugs in exerting better therapeutic effects. In a recent study, Monteros et al. found the oral administration of *L. casei* CRL 431 and *L. paracasei* CCM I-1518 ameliorated the indomethacin-induced inflammatory responses by macrophages by reducing ROS and pro-inflammatory cytokine production [113]. Sharaf et al. proved that the preventive treatment of LGG or/and *L. acidophilus*, combined with celecoxib, could better exert an anti-tumor effect by up-regulating pro-apoptotic BAX and down-regulating anti-apoptotic BCL-2 protein [114]. Mi et al. found that *Bifidobacterium infantis* enhanced the anti-tumor effect and alleviated 5-FU-induced mucositis by inhibiting the Th1 and Th17 responses and promoting the Foxp3 Treg responses in CRC rats [115]. These studies, taken together, illustrate that probiotics can activate the immune system to kill cancer cells and relieve drug-induced tumor suppression, thereby playing the role of adjuvant drugs. Regulation of the immune system is an important mechanism by which probiotics work.

### 4.5. The Interaction of Multiple Mechanisms

The probiotic effect of probiotics on the body is often mediated by multiple mechanisms. Specifically, after arriving in the gut, probiotics regulate the intestinal microenvironment by changing the intestinal pH and grabbing nutrition, thus increasing the relative abundance of intestinal probiotics and promoting the secretion of beneficial metabolites to alleviate drug damage [116]. For example, SCFAs are well-known intestinal metabolites that are up-regulated by probiotics, which can alleviate drug-induced damage [117]. In addition, probiotic supplementation can inhibit the growth of harmful bacteria in the intestinal tract, maintain the intestinal barrier and alleviate drug-induced intestinal inflammation, thereby inhibiting the exudation of inflammatory factors so as to prevent the transfer of inflammation and regulate the secretion and differentiation of immune cells, and regulate the immune system to resist the side effects of drugs [73]. Probiotics can also intervene in the vicious circle of oxidative stress and inflammation by eliminating excessive free radicals and regulating the activity of oxidase to avoid unnecessary internal friction in the immune system. There are more unknown mechanisms for probiotics. Therefore, the puzzle of probiotics is like the stars in the universe, and we still need to continue our exploration.

## 5. Conclusions

Due to the changes in our modern living environment and dietary habits, the types and incidence of diseases have increased, resulting in the use of drugs becoming gradually rampant, which in turn led to the problems of side effects and a decreased efficacy of drugs. By summarizing these types of side effects and integrating the changes in the intestinal flora, we propose targeting the intestinal flora, which may be used to assist the use of drugs, which may improve the efficacy of drug treatment while reducing the risks. Probiotics have been confirmed by many pieces of literature to resist the side effects of drugs, including DILI, DIKI, chemotherapy side effects and so on. Probiotics can also be used as an engineering carrier to deliver part of the drug components to the diseased site so that it can play a better role. According to the resistance to drug side effects and the improvement of drug efficacy and its potential mechanism summarized in this paper, it can be concluded that probiotics will be better valued in clinical practice as auxiliary drugs in the future.

## Figures and Tables

**Table 1:** Micro-organisms used as probiotics.

Bacteria [14,15]				Yeast and Molds [16]
*Lactobacillus* spp.	*Bifidobacterium* spp.	*Streptococcus* spp.	Others	
*L. acidophilus* *L. sporgenes* *L. plantarum* *L. rhamnosum* *L. delbrueck* *L. reuteri* *L. fermentum* *L. lactus* *L. cellobiosus* *L. brevis* *L. casei* *L. farciminis* *L. paracasei* *L. gasseri* *L. crispatus*	*B. bifidum* *B. infantis* *B. adolescentis* *B. longum* *B. thermophilum* *B. breve* *B. lactis* *B. animalis*	*S. lactis* *S. cremoris* *S. alivarius* *S. intermedius* *S. thermophilis* *S. diacetylactis*	*Leuconostoc mesenteroides* *Pediococcus* *Propionibacterium* *Bacillus* *Enterococcus* *Enterococcus faecium*	*Saccharomyces cerevisiae* *Saccharomyces boulardii* *Aspergillus niger* *Aspergillus oryzae* *Candida pintolopesii*

**Table 2:** Recent studies on gut microbiome in DILI treated with probiotics.

Author/Date	Medicine	Method (The Probiotic)	Changes in Intestinal Microbiome in Probiotic Intervention
Chen et al. [43]	*α*-naphthylisothiocyanate and valproate acid	encapsulating probiotic *Lactobacillus delbrueckii* subsp. *Bulgaricus* and *Lactobacillus rhamnosus* GG into Ca^2+^-complexed polymer microspheres	↑*Lactobacillus*, *Colidextribacter*, *Allobaculum*, *Enterorhabdus*, *Bifidobacterium*, *Gordonibacter* of Actinobacteria ↓*Muribaculum* and *Desulfovibrio*
Yu et al. [44]	D-Galactosamine	*Saccharomyces boulardii*	↑*Bacteroidaceae* and *Clostridiaceae* ↓*Alcaligenaceae*, *Anaeroplasmataceae*, *Caulobacteraceae* and *Rikenellaceae*
Xia et al. [45]	APAP	*Akkermansia muciniphila* Muc ^T^ ATCC BAA-835	↑*Lactobacillus*, *Candidatus_Saccharimonas* and *Akkermansia* ↓*Oscillibacter*, *Colidextribacter*, *Pseudaminobacter*, *Ruminiclostridium* and *Idiomarina*
Wu et al. [46]	Diquat	*Bacillus amyloliquefaciens* SC06, *Bacillus licheniformis* SC08	↑*Anaerofilum*, *Bacteroides* uniformis, *Helicobacter* ↓*Oscillospira guilliermondi*
Gu et al. [47]	Alcohol	*Lactobacillus rhamnosus GG* granules	↑*Lactobacillus* and *Bifidobacterium* ↓*Clostridium perfringens*, *Proteobacteria*, *Campylobacterales *and* Helicobacter*
Liu et al. [48]	CCl_4_	*Clostridium butyricum*	↑*Lactobacillales*, *Clostridiales*, *Erysipelotrichales* ↓*Bacteroidales*
Tian et al. [49]	Alcohol	*Lactobacillus rhamnosus* CCFM1107	↑*Lactobacilli*, *Bifidobacteria* ↓*Enterococci*, *Enterobacter*

**Table 3:** Recent studies on the efficacy of probiotics in alleviating side effects of chemotherapy.

Author/Date	Chemotherapy Drugs	Method (The Probiotic)	Effect
Castelli et al. [66]	Paclitaxel	A bacterial extract of nine probiotics	Relieving chemotherapy-induced neuropathic pain
Reyna-Figueroa et al. [67]	Prednisone, vincristine, daunorubicin and L-asparaginase	*L. rhamnosus* GG	Reducing gastrointestinal side effects (nausea, vomiting and bloating)
do Carmo et al. [68]	5-FU	*Propionibacterium freudenreichii* CIRM-BIA 129	Alleviating mucositis by secreting SlpB protein
Xia et al. [69]	Cisplatin	Probiotic cocktail made from *L. plantarum*, *Bifidobacterium animalis*, *L. rhamnosus* and *L. acidophilus*	Reducing the severity of oral mucositis induced by modulating gut microbiota homeostasis and enhancing host immunity
Zhang et al. [70]	Epirubicin plus cyclophosphamidum, Epirubicin plus cyclophosphamidum combined with docetaxel or docetaxel plus cyclophosphamidum	Probiotic capsule contained *Bifidobacterium longum*, *L. acidophilus* and *Enterococcus faecalis*	Improving overall cognitive function in patients, altering gut microbial composition and increasing plasma levels of p-Mentha-1,8-dien-7-ol to prevent cognitive impairment in breast cancer patients
Wu et al. [71]	Cisplatin	A mixture of probiotics (*Bifidobacterium breve*, *L. acidophilus*, *L. casei* and *S. thermophilus*)	Improving mucositis and pica by modulating intestinal flora and inhibiting 5-hydroxytryptamine secretion
Yuan et al. [72]	Oxaliplatin	BIO-THREE tablets, made from *C. butyricum* TO-A, *Bacillus mesentericus* TO-A and *Streptococcus faecalis* T-110	Restoring the abundance of *Bacteroides* and *Prevotella*, then attenuating intestinal damage
Ren et al. [73]	CPT-11	*Bifidobacterium animalis* subsp. *lactis* SF	Increasing the relative abundance of anti-inflammatory bacteria, inhibiting intestinal inflammation caused by CPT-11, protecting intestinal barrier to inhibit the leakage of TGF- β, thereby inducing apoptosis and autophagy of tumor cells and alleviating CPT-11-mediated immunosuppression
Cuozzo et al. [74]	Paclitaxel	Probiotic formulation SLAB51	Alleviating peripheral neuropathy by increasing the expression of opioid and cannabinoid receptors in the spinal cord, preventing the reduction of nerve fiber damage in the paw, and modulating serum pro-inflammatory cytokine concentrations

## Data Availability

Not applicable.
